# Numerical Analysis on the Excavation Damage Evolutions of Layered Tunnels: Investigations on the Influences of Confining Pressure and Layer Angles

**DOI:** 10.3390/ma17215266

**Published:** 2024-10-29

**Authors:** Wangping Qian, Xu Tang, Shuyang Yu, Xing Li, Yuexin Chen

**Affiliations:** 1 School of Mechanics and Civil Engineering, China University of Mining and Technology, Xuzhou 221116, China; qianwangping@my.swjtu.edu.cn (W.Q.); ts24030131a31@cumt.edu.cn (X.T.); 2 School of Transportation and Civil Engineering, Nantong University, Nantong 226019, China; 3 Key Laboratory of Transportation Tunnel Engineering Ministry of Education, Southwest Jiaotong University, Chengdu 610031, China; 4 Nanjing Hydraulic Research Institute, Nanjing 210029, China; xingli@nhri.cn; 5 College of Civil Engineering, Nantong Institute of Technology, Nantong 226001, China; chenyx_hhu@163.com

**Keywords:** tunnel excavation, bedding structures, stress boundary, numerical simulation

## Abstract

The bedding structure of layered tunnels has a significant impact on the evolution of excavation damage, yet research on the relevant evolution mechanisms is scarce. In view of this, this paper develops a mesh-free numerical method to simulate the progressive damage process of tunnel excavation and proposes a method for applying stress boundaries within the SPH framework. Through this method, simulations of tunnel excavation damage under different bedding dip angles and stress ratios are conducted. The results show that the following: in the simulation of excavation damage of a tunnel without bedding structures, specific areas around the tunnel exhibit characteristics of tensile–shear composite failure and shear failure, verifying the rationality of the algorithm; under different bedding dip angles, a damage zone is first generated around the tunnel, and shear cracks appear at the tangent of the bedding plane and the tunnel, with the damage degree being the largest when α = 30° and the smallest when α = 45°; and under different stress ratios, the damage starts around the tunnel, continuously evolves, and finally forms a failure zone inside the bedding plane joints tangent to the tunnel, and the damage degree increases with the increase in the stress ratio. This study discusses the damage mechanisms under different calculation schemes and provides a reference for understanding the excavation damage mechanisms of layered tunnels.

## 1. Introduction

With the rapid development of the social economy, people′s demand for water resources is also increasing. In order to meet the needs of water supply, irrigation, and power generation, more and more hydraulic tunnels need to be built to transport and deploy water resources [1,2]. Hydraulic tunnels are underground passage structures specifically designed and constructed for water conservancy projects. They serve as an effective means of water resource allocation and can mitigate damage and pollution to the natural environment, fulfilling the requirements of sustainable development [3,4,5]. Most hydraulic tunnels in China are distributed in high mountain and canyon areas in the southwest. Many of them have reached a depth of dozens of meters or even several hundred meters or more [6,7]. The construction of tunnels exhibits a trend towards full-face excavation and large-area unloading. The excavation of tunnels leads to changes in the stress field of the surrounding rocks. The change in stress also causes changes in the rock mass structure. Moreover, they restrict and influence each other, and the interaction process is quite complex. Therefore, mastering the excavation damage evolution mechanisms and damage range of tunnels under complex loads and conditions will undoubtedly provide important guiding roles for the support, operation, and safety protection of hydraulic tunnels.

Research on tunnel excavation damage mechanisms mainly focuses on three aspects: experimental research, theoretical research, and numerical simulation research. Experimental research can intuitively show the excavation damage range and fracture morphology of tunnels and is the basis of theoretical research. For example, Lei et al. [8] conducted in-depth research on the interaction mechanisms between surrounding rocks and linings in shallow-buried and unsymmetrically loaded tunnels. They particularly focused on the influence of different deflection angles on the test results, that is, how the inclination or slope of the tunnel affects the interaction between surrounding rocks and linings. Yu et al. [9] used a fluid–solid coupling model test to explore the influence of seepage on the mechanical behavior of surrounding rocks. Their research results show that indoor model tests can better consider factors such as surrounding rock weakening caused by seepage and are closer to actual engineering situations. Therefore, they are more valuable for solving practical engineering problems. Sheng et al. [10] used a plane strain model to simulate the entire process of tunnel excavation and support in inclined upper-soft and lower-hard strata. They studied in detail the deformation laws of surrounding rocks and the change in lining internal forces during the construction processes, and they particularly emphasized that in inclined composite strata, close attention needs to be paid to the asymmetric deformation problem of surrounding rocks. Zhang et al. [11] used a plane strain model test system to conduct in-depth research on the construction situation of tunnels in composite strata. They particularly focused on the influence of the location, thickness, and relative stiffness of weak strata on the interaction between surrounding rocks and support structures. Li et al. [12,13] successfully developed a new fluid–solid coupling simulation test system, which is used to simulate the interaction between fluids (such as groundwater) and solids (such as surrounding rocks and lining structures) in underground engineering. However, experimental research can only obtain the apparent morphology of tunnel excavation damage; it is difficult to reflect the internal fracture mechanisms of tunnel excavation damage. Theoretical research is based on experimental research phenomena and summarizes as well as deduces mathematical formulas for the excavation damage range of tunnels or fracture damage criteria for a tunnel’s surrounding rock damage. For instance, Chen et al. [14] put forward an important mathematical expression to elucidate the correlation between the support reaction force on the tunnel wall and the convergence displacement of the tunnel wall. The derivation of this expression is based on the Mohr–Coulomb strength criterion and ideal elastoplastic theory and can be used to understand and predict the behavior of support structures in the tunnel convergence process more deeply. Carranza-Torres et al. [15] constructed the characteristic curve of surrounding rocks of circular roadways in ideal elastoplastic rock masses under hydrostatic stress in their research and deeply discussed its joint application with the support characteristic curves of common support structures in underground engineering. Carranza-Torres and Fairhurst [16] focused on discussing the relationship between the empirical parameters in the Hoek–Brown criterion and the characteristic curve of surrounding rocks. Their research mainly focuses on the ideal elastoplastic model, which provides important support for understanding the mechanical behavior of surrounding rocks in underground engineering. Sharan [17,18] pointed out some inadequacies in the analytical solution proposed by Brown and further proposed an approximate analytical solution based on an ideal elastic–brittle–plastic model. However, theoretical research can only obtain analytical solutions under simple boundaries and geometric shapes; is difficult to analyze complex fracture morphologies and boundary condition situations.

Numerical simulation can overcome the deficiencies of experimental research and theoretical research. It can not only simulate tunnel excavation damage problems under complex geometric shapes and boundary conditions but it can also reflect the internal fracture mechanisms that cannot be shown in experimental research. Therefore, it is hailed as the “third means” of scientific research. The finite element method is the earliest numerical method applied to tunnel excavation damage simulation. For example, Liu et al. [19] studied the seismic failure mechanism of mountain tunnels based on the IBEM-FEM (Indirect Boundary Element Method-Finite Element Method) coupling method. Xie et al. [20] established a three-dimensional nonlinear brittle elastoplastic mechanical model based on FEM and analyzed the mechanism of surrounding rock stability and instability during tunnel excavation. Zhuang et al. [21] established a finite element model of adjacent tunnels based on FEM and analyzed the displacement changes of adjacent tunnels caused by tunnel excavation. However, in the process of dealing with discontinuous problems such as tunnel excavation damage, the finite element method requires mesh refinement at the crack locations. At the same time, in the process of damage evolution simulations, the mesh needs to be redivided at all times, resulting in a large amount of calculation. Meanwhile, for complex fracture expansion situations, the mesh division is extremely distorted, even showing mesh division failure, affecting the calculation accuracy [22]. The discrete element method is a meshless method different from the finite element method. This method discretizes the calculation domain of the model into a series of particles and endows the particles with corresponding contact properties so that it can simulate typical discontinuous problems such as tunnel excavation damage. For example, Wang et al. [23] used the spherical discrete element model (DEM) and a new flexible membrane technology to achieve transient loading of three principal stresses of any size and direction and simulated the stress–strain response under transient unloading in tunnel excavation. Zhou et al. [24] proposed an FDM-REV-DEM interface coupling method and applied the coupling method to the Shantou Bay subsea tunnel of the Shantou–Shanwei high-speed railway based on the concept of representative elementary volume (REV). Liu et al. [25] proposed a numerical method for predicting the EDZ under coupled THM (Thermo–Hydro–Mechanical) conditions based on the discrete element method (DEM) and studied the influence of coupled THM (Thermo–Hydro–Mechanical) conditions on the characteristics of EDZ (Excavation Damaged Zone). However, the discrete element method has many mesoscopic parameters. Complex parameter calibration is required before numerical simulation. The determination of its value largely depends on the experience [26]. In recent years, many popular numerical simulation methods such as peridynamics (PD) [27,28], numerical manifold method (NMM) [29,30], and phase field method (PF) [31,32] have been proposed. However, these numerical methods still have some problems. For example, the Poisson′s ratio in the bond-based peridynamics method is a constant, which is inconsistent with the real situation. The crack of the numerical manifold method must fall on the mesh nodes. The phase field method still needs to divide the mesh, which is more difficult to use to simulate the interaction of complex crack propagation problems. The smoothed particle hydrodynamics method (SPH) is a Lagrangian meshless method that can better adapt to the discontinuous characteristic of the rock fracture processes. For example, the GPD (General Particle Dynamics) method proposed by Zhou et al. [33,34,35,36,37,38,39,40,41,42,43,44] based on the SPH method has a good application in the simulations of the progressive failure process of rock crack propagations. However, the SPH method has been rarely applied in the study of bedding tunnel excavation damage simulations.

Based on the deficiencies of previous studies, this paper introduces a fracture marker *к* that characterizes the failure state of particles, which can realize the simulation of tunnel excavation damage under the SPH framework. At the same time, stress particles under the SPH framework are introduced to realize the application of confining pressure. The introduction of “live and dead particles” can realize the removal of tunnel particles and thus realize the simulation of tunnel excavation. The tunnel excavation damage simulations under different bedding inclination angles and different stress ratios are carried out. The research results can provide a reference for understanding the bedding tunnel excavation damage mechanisms and the applications of the SPH method in the prediction and simulation of tunnel engineering disasters.

## 2. Principles and Improvement Methods of SPH

### 2.1. Basic Property Equations of SPH

For SPH calculation, kernel function approximation and particle approximation should be performed first. Among them, the kernel function approximation method approximates the values and derivatives of continuous field quantities through discrete sampling points (particles), and its expression can be written as

(1) $$f(x)\approx \int_{\Omega }f(x^{\prime })W(x-x,\;h)dx$$

where *x* is the particle coordinate vector; *f* represents the field quantity function and is used to represent variables such as density and velocity; *Ω* is the calculation domain of SPH; and *W* represents the smooth kernel function under the SPH framework.

The particle approximation method is a further approximation of the kernel approximation equation which uses discretized particles in SPH. By applying the superimposed summation of the corresponding values of adjacent particles in the local area to replace the integral of the field function and its derivative, its expression can further rewrite Equation (1) and can be expressed as

(2) $$f(x_{i})=\sum_{j=1}^{N}\frac{m_{j}}{\rho_{j}}f(x_{j})•W_{ij}\;$$

In Equation (2), *m* is the mass of the particle and *ρ* is the density of the particle.

During the SPH calculation process, the parameters between particles are transferred through the control equation to update the parameters of the particles. The key control equations of SPH include the continuity equation and the momentum equation, and their expressions can be written as [45]

(3) $$\frac{d\rho_{i}}{dt}=\sum_{j=1}^{N}m_{j}v_{ij}^{\beta }\partial W_{ij,\beta }$$

(4) $$\frac{dv_{i}^{\alpha }}{dt}=\sum_{j=1}^{N}m_{j}(\frac{\sigma_{ij}^{\alpha \beta }}{\rho_{i}^{2}}+\frac{\sigma_{ij}^{\alpha \beta }}{\rho_{j}^{2}}+T_{ij})\partial W_{ij,\beta }$$

Among them, *v* and *σ* are the velocity and stress of the particle; the subscripts *i* and *j* of the parameters, respectively, represent the particle serial number; *t* represents the time parameter; *N* is the total number of SPH particles; and *α* and *β* are Einstein notations.

### 2.2. Numerical Treatment Method for Particle Failure in SPH

In this section, the Mohr–Coulomb criterion is used to determine whether one particle fails. First, it is determined whether the maximum principal stress *σ*_1_ of the particle reaches its tensile strength *σ_t_*. If it is satisfied, the particle fails. If it is not satisfied, it is then determined whether the particle undergoes shear failure. The expression of the Mohr–Coulomb criterion is as follows [46]:

(5) $$\sigma_{1}=\sigma_{t}$$

(6) $$\tau_{f}=c+\sigma_{f}\tan\varphi$$

Among them, *c* is the cohesion of SPH particles; and *σ_f_* and *τ_f_* are the normal stress and shear stress on the material failure surface, respectively.

**Figure 1:**
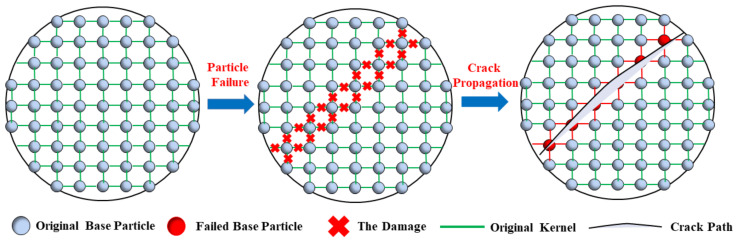
Numerical processing process of SPH particle failure.

It can be found from the control Equations (3) and (4) in SPH that the derivative of the kernel function controls the parameter transfer between SPH particles. Therefore, in this section, a parameter *к* that can characterize whether a particle fails is introduced. When the particle is not damaged, set *к* = 1. When the particle reaches the failure criterion (5) or (6), set *к* = 0, indicating that the particle is damaged. The numerical processing flow of the particle failure processes under the SPH framework is shown in Figure 1. Multiplying the fracture marker *к* by the traditional smooth kernel function *W* can obtain the expression of the improved kernel function *I* considering particle failure:

(7) $$I(x-x^{\prime },\;h)=\kappa \cdot W(x-x^{\prime },\;h)$$

Replacing the traditional smooth kernel function *W* with the improved kernel function *I* can obtain the SPH control equation considering particle failure:

(8) $$\frac{d\rho_{i}}{dt}=\sum_{j=1}^{N}m_{j}v_{ij}^{\beta }\partial I_{ij,\beta }$$

(9) $$\frac{dv_{i}^{\alpha }}{dt}=\sum_{j=1}^{N}m_{j}(\frac{\sigma_{ij}^{\alpha \beta }}{\rho_{i}^{2}}+\frac{\sigma_{ij}^{\alpha \beta }}{\rho_{j}^{2}}+T_{ij})\partial I_{ij,\beta }$$

### 2.3. Application Method of SPH Stress Boundary

**Figure 2:**
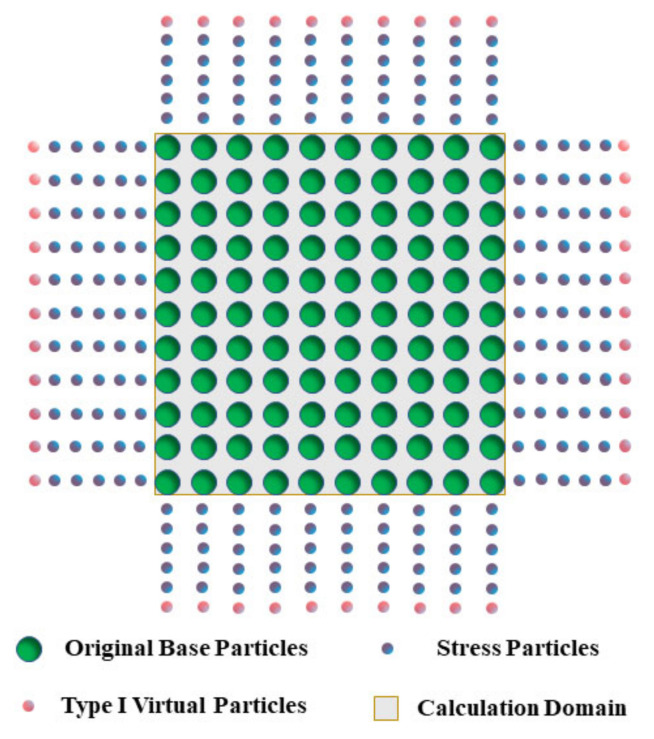
Application method of stress boundary under the SPH framework.

The stress boundary of SPH adopts the stress mapping method, and “stress particles” of more than five layers are arranged outside the original base particles, as shown in Figure 2. At the same time, “stress particles” need to have the following characteristics:

(1) “Stress particles”, like original base particles, participate in the calculation of control equations to transfer characteristic parameters.
(2) For each calculation step, stress is reassigned to “stress particles”. That is, although “stress particles” participate in the parameter transfer of original base particles, their stress changes meet the preset stress boundary requirements.
(3) Outside the stress particles, an additional layer of “type I virtual particles” needs to be arranged, and their initial velocity is set to 0.

### 2.4. Simulation Method of SPH Tunnel Excavation

In this section, the concept of “live and dead particles” under the SPH framework is introduced, and “tunnel particles” are predefined in the modeling process. During the application process of initial confining pressure, set the fracture marker *к* of “tunnel particles” to 1. At this time, “tunnel particles”, like original base particles, participate in the calculation of SPH control equations and the transfer of parameters. When performing the tunnel excavation operation, “tunnel particles” are killed at this time, the fracture marker *к* of “tunnel particles” is set to 0, and “tunnel particles” are hidden and displayed. At this time, the simulation of tunnel excavation is realized.

## 3. Analysis of Simulation Results

### 3.1. Numerical Model and Parameters

**Figure 3:**
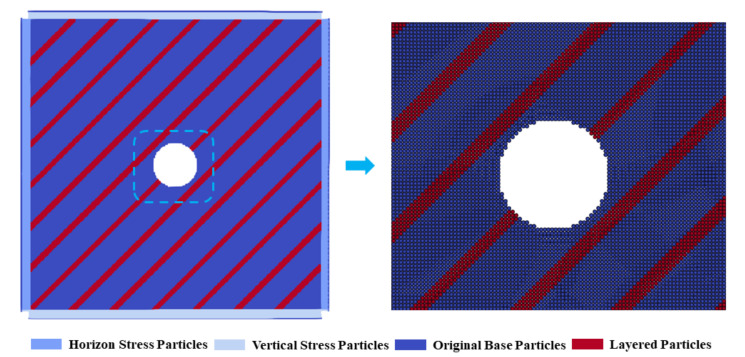
Numerical model and particle subdivision.

Bedding refers to the structural surfaces with layered characteristics formed in the rock due to differences in material composition, particle size, color, etc., during the sedimentary process. These bedding planes are usually arranged approximately parallel to each other and have relative continuity within a certain area (as shown in Figure 3). During the tunnel excavation process, the presence of bedding planes will change the stress propagation path, making the stress distribution around the tunnel more complex. Compared with faults, bedding planes do not have obvious dislocation and displacement, but their influence on the tunnel excavation damage is equally significant. For example, different bedding dip angles (*α*) will lead to significant differences in the stress release and damage evolution patterns during tunnel excavation, which will be discussed in detail in the subsequent sections.

Figure 3 shows the size and particle subdivision of the tunnel excavation model with bedding planes. Among them, the model size is taken to be more than five times the tunnel diameter, which is 100 m × 100 m. A circular excavation body with a radius of 6.2 m is set in the middle of the model. Corresponding confining pressure is applied to the model boundary. The entire model is divided into 200 × 200 = 40,000 particles. The numerical stability of the method has been proved by previous studies. The parameters of the model are as follows [47]: for the rock mass matrix, the elastic modulus *E* = 50.59 Mpa, the tensile strength *σ_t_* = 11.523 MPa, and the Poisson′s ratio *μ* = 0.14; the cohesion *c* = 23.95 MPa, and the internal friction angle *φ* = 40°; for the bedding structure, the elastic modulus *E* = 10 Mpa, the tensile strength *σ_t_* = 7.5 MPa, and the Poisson′s ratio *μ* = 0.14; the cohesion *c* = 18.895 MPa, and the internal friction angle *φ* = 40°. The model gradually applies confining pressure within the initial 7000 steps. The tunnel excavation operation is performed at 7000 steps, and the calculation continues until 10,000 steps.

To explore the influence of different bedding dip angles (defined as the angle *α* between bedding and the horizontal direction) and different stress ratios (defined as the ratio of horizontal confining pressure *σ_h_* to vertical confining pressure *σ_H_*, for the fact that the Jinping deep tunnel has various stress ratio ranges) on the damage range of tunnel excavation, the following calculation schemes are set: Scheme A: different bedding included angles are *α*, A1: *α* = 15°, A2: *α* = 30°, A3: *α* = 45°, A4: *α* = 60°; Scheme B: different stress ratios are *σ_h_*/*σ_H_*, B1: *σ_h_*/*σ_H_* = 0.7, B2: *σ_h_*/*σ_H_* = 0.8, B3: *σ_h_*/*σ_H_* = 0.9, B4: *σ_h_*/*σ_H_* = 1.

### 3.2. Model Verification

**Figure 4:**
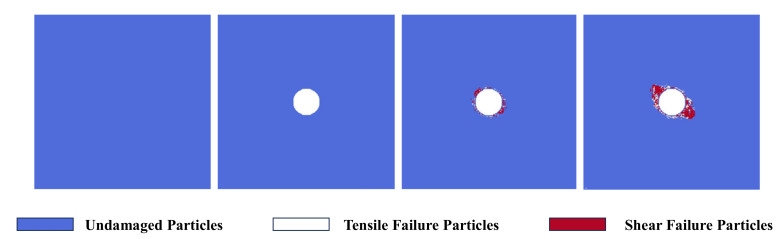
The excavation damage process of tunnel in the model without bedding.

**Figure 5:**
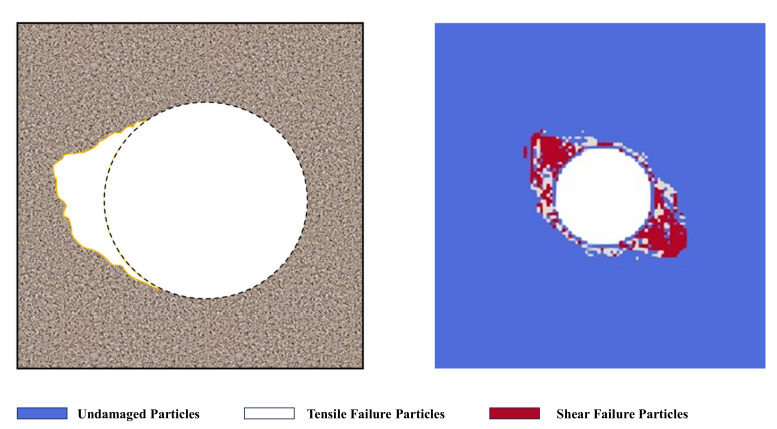
Comparison of actual engineering and numerical simulation results of tunnel excavation damage.

To verify the rationality of the numerical simulation in this paper, Figure 4 and Figure 5 show the excavation damage process of the tunnel model without bedding structure and the comparison with the previous engineering damage and failure. Among them, the white marked particles represent tensile failure particles, and the red marked particles represent shear failure particles. As can be seen from the figure, with the tunnel excavation operation, an excavation damage area appears around the tunnel. Among them, tensile and shear failure particles mainly appear in the two o′clock and eight o′clock directions around the tunnel, indicating that tensile–shear composite failure mainly occurs here. In the 4 o′clock and 10 o′clock directions around the tunnel, shear failure particles mainly appear, indicating that shear failure mainly occurs here. And as the loading steps continue, the damage area in the 4 o′clock and 10 o′clock directions around the tunnel becomes larger, indicating that excavation failure occurs here. It can be seen from Figure 5 that the excavation damage morphology of the tunnel model without bedding structure in this paper is highly consistent with the excavation damage range of the previous Jinping tunnel [44], which verifies the rationality of the numerical simulation in this paper.

### 3.3. Influence of Different Bedding Dip Angles on Tunnel Excavation Damage

**Figure 6:**
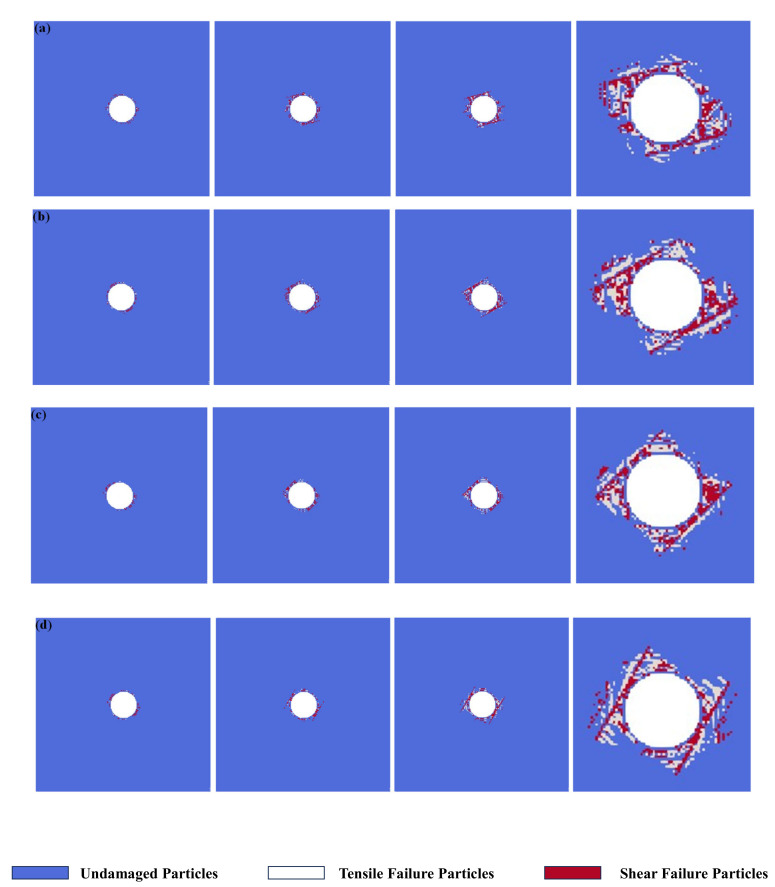
Simulation results of tunnel excavation damage under different bedding dip angles. (**a**) *α* = 15°; (**b**) *α* = 30°; (**c**) *α* = 45°; (**d**) *α* = 60°.

Figure 6 presents the numerical simulation results of the excavation damage process of bedding tunnels under different bedding dip angles. As can be seen from the figure, the existence of bedding structure greatly changes the excavation failure modes of tunnels. For the bedding dip angle *α* = 15°, with the tunnel excavation operation, a circle of excavation damage zones is first generated around the tunnel. However, it should be noted that obvious shear cracks are generated at the place where the bedding is tangent to the tunnel (the red area in Figure 6a). As the loading steps continue, the excavation damage zone gradually develops along the bedding direction, and the cracks generated along the bedding are mostly shear cracks. When the excavation damage develops along the bedding plane to a certain extent and then stops expanding, a through vertical crack is formed between the bedding layers. Its direction is approximately perpendicular to the bedding structure, and the cracks generated between the bedding layers are tensile–shear composite cracks. Finally, the damage expands around the bedding structure, causing the excavation failure of the tunnel. For the bedding dip angle *α* = 30°, the damage evolutions around the tunnel are the same as the circumstance of *α* = 15°. First, shear cracks are generated at the position where the joint is tangent to the tunnel. Then, the difference from *α* = 15° is that obvious crack propagation occurs at the two beddings tangent to the tunnel. After the cracks of the two dominant joints expand to a certain extent, more excavation damage zones are generated between the two beddings, which comprise tensile–shear composite failure. Finally, the large-scale excavation damage caused by the two dominant beddings leads to the failure of the tunnel model. For the bedding dip angle *α* = 45°, after tunnel excavation, obvious shear crack propagation occurs in the 5 o′clock and 11 o′clock directions around the tunnel. As the calculation steps continue, two dominant beddings tangent to the tunnel generate crack propagation. Subsequently, a large amount of tensile–shear composite failure occurs between the two dominant beddings. It should be noted that at this time, under the same calculation steps, the damage degree of the whole model is the smallest. For the bedding dip angle *α* = 60°, obvious shear crack propagation occurs in the 4 o′clock and 10 o′clock directions around the tunnel. As the calculation steps proceed, two dominant beddings with a 60° dip angle tangent to the tunnel generate obvious crack propagation. As the calculation steps continue, the cracks at the two dominant beddings continuously expand, driving the generation of a large number of excavation damage zones around them, thus leading to the failure of the model.

### 3.4. Influence of Different Stress Ratios on Tunnel Excavation Damage

**Figure 7:**
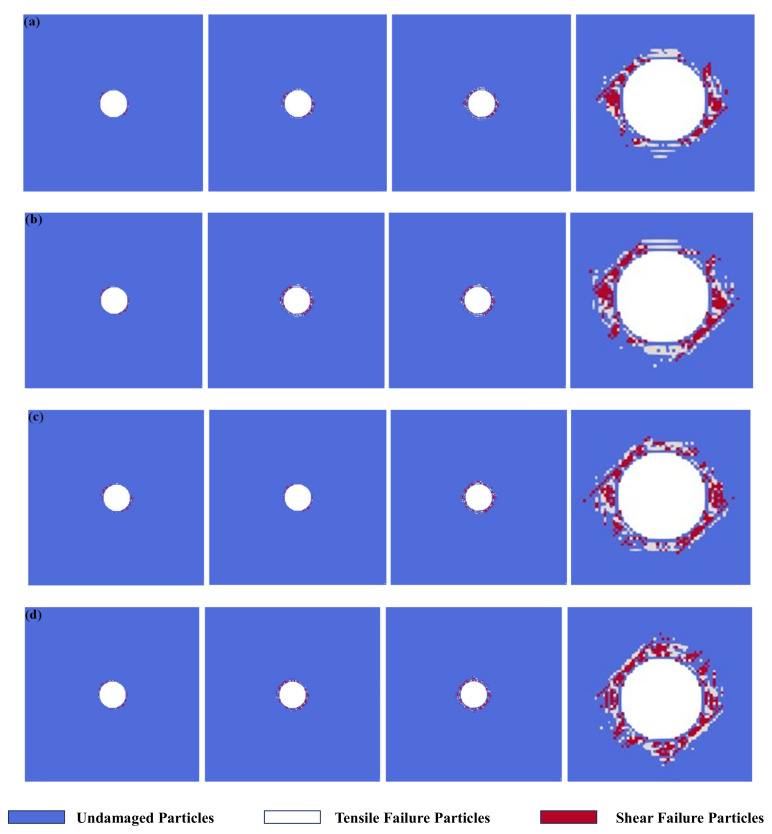
Numerical simulation results of tunnel excavation damage under different stress ratios. (**a**) *σ_h_*/*σ_H_* = 0.7; (**b**) *σ_h_*/*σ_H_* = 0.8; (**c**) *σ_h_*/*σ_H_* = 0.9; (**d**) *σ_h_*/*σ_H_* = 1.

Figure 7 presents the numerical simulation results of the excavation damage process of bedding tunnels under different stress ratios. As can be seen from the figure, the evolution modes of tunnel excavation damage under different stress ratios are relatively similar. First, damage is generated around the tunnel. Then, the damage continuously evolves. Finally, a damage zone surrounding the tunnel is formed on the inner side of the two joints tangent to the tunnel, leading to the failure of the model. However, the final tunnel excavation damage ranges are different under various stress ratios. With the increase in stress ratio, the concentrated area of excavation damage gradually shifts from the three o′clock and nine o′clock directions around the tunnel to being concentrated around the tunnel. The damage degree also shows a gradually increasing rule with the increase in stress ratio.

### 3.5. Variation Laws of Damage Count Under Different Calculation Schemes

**Figure 8:**
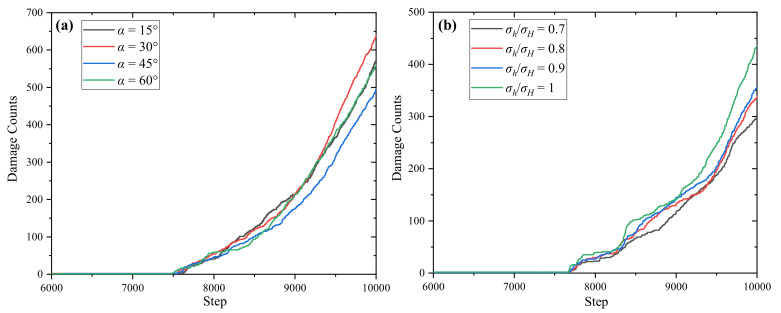
Variation laws of damage count with calculation steps under different calculation schemes. (**a**) Different bedding dip angle schemes; (**b**) different stress ratio schemes.

Figure 8 presents the variation laws of damage count under different calculation schemes. As can be seen from the figure, before 7000 calculation steps, there is no crack propagation. At the 7000th calculation step, the tunnel is excavated and the stress in the tunnel is released. Damage begins to occur at step 7500. After tunnel excavation, the damage increases sharply with the calculation steps, indicating that the destruction after tunnel excavation is relatively rapid. For different joint dip angles, the damage degree is the largest when *α* = 30° and the smallest when *α* = 45°. For different stress ratios, with the increase in stress ratio, the excavation damage degree of the tunnel gradually increases.

## 4. Discussion

### 4.1. Excavation Damage Mechanism of Tunnels Under Different Bedding Dip Angles

**Figure 9:**
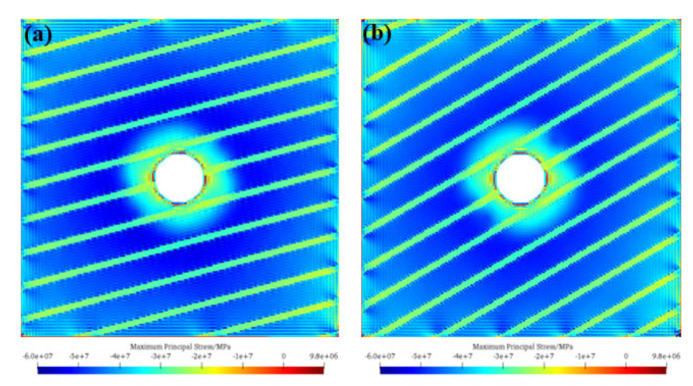

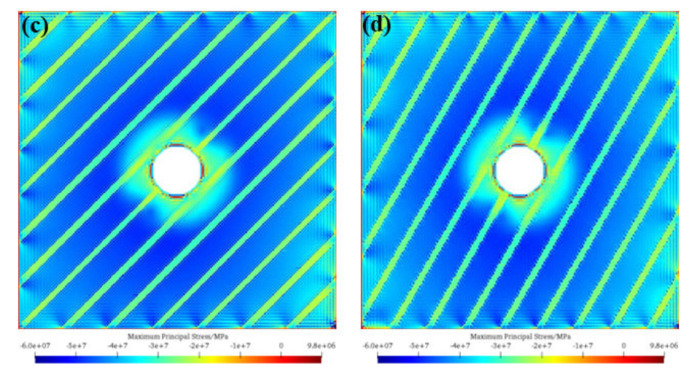
Maximum principal stress nephogram after tunnel excavation under different bedding dip angles. (**a**) *α* = 15°; (**b**) *α* = 30°; (**c**) *α* = 45°; (**d**) *α* = 60°.

Figure 9 presents the maximum principal stress nephogram after tunnel excavation under different bedding dip angles. As can be seen from the figure, after tunnel excavation, stress is released. The compressive stress around the tunnel is significantly less than that inside the surrounding rock. At the same time, a small range of relatively high tensile stress concentration is generated around the tunnel, which is also the reason for the excavation damage around the tunnel. For *α* = 15° and *α* = 30°, the stress release range around the tunnel is large, so the final tunnel excavation damage degree is also large. For *α* = 45° and *α* = 60°, at the joint passing through the center of the tunnel, there is an obvious shrinking trend in the stress release range, indicating that the stress release degree is small at this time. Therefore, the damage degree of these two cases is slightly less than that of *α* = 15° and *α* = 30°.

### 4.2. Excavation Damage Mechanism of Tunnels Under Different Stress Ratios

**Figure 10:**
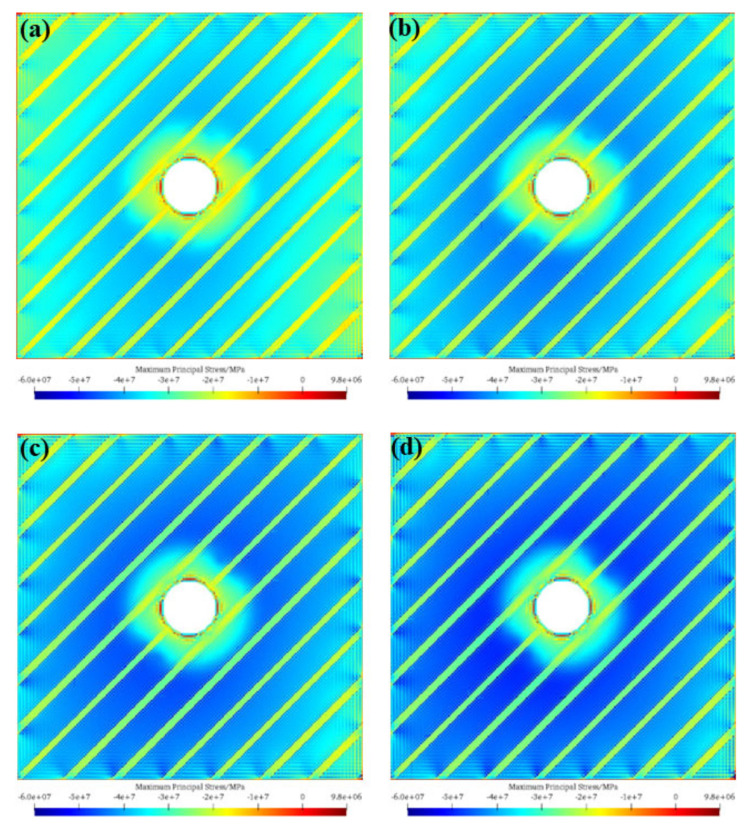
Maximum principal stress nephogram after tunnel excavation under different stress ratios. (**a**) *σ_h_*/*σ_H_* = 0.7; (**b**) *σ_h_*/*σ_H_* = 0.8; (**c**) *σ_h_*/*σ_H_* = 0.9; (**d**) *σ_h_*/*σ_H_* = 1.

Figure 10 presents the maximum principal stress nephogram after tunnel excavation under different stress ratios. As can be seen from the figure, under different stress ratios, the stress release zones around the tunnel have similar shapes. However, as the stress ratio increases, the range of the stress release zone becomes smaller, but the degree of stress release becomes larger. Therefore, as the stress ratio increases, the degree of tunnel excavation damage gradually increases.

### 4.3. Limitations and Future Plans of This Study

In the context of this study, the assumption of homogeneity was made primarily to isolate and focus on the specific effects of bedding angles and stress ratios on tunnel excavation damage. By initially considering a relatively simplified model, we were able to develop and validate the numerical simulation framework, including novel techniques such as the fracture marker к, stress particle application, and “live and dead particle” concept for tunnel excavation simulation. However, real-world rock masses are heterogeneous and exhibit significant heterogeneity in material properties due to various factors such as geological formation processes, mineral composition variations, and the presence of fractures and discontinuities. This heterogeneity can have a profound impact on the behavior of tunnels during excavation and the evolution of damage. Future studies should focus on incorporating more realistic representations of material heterogeneity in the model by conducting detailed geological surveys and laboratory tests to obtain data on the spatial variability of rock properties within the bedding structures. Meanwhile, the monitoring of real-world tunnel engineering will be of great significance [48].

### 4.4. Discussions About the Broader Applicability of This Method

Although the current research mainly focuses on the simulation of tunnel excavation damage under specific bedding structures and stress ratios, the numerical simulation method adopted has the potential to be extended to other geological settings and tunnel types to some extent: (1) In terms of geological material properties, although this study assumes the homogeneity of rock materials, the properties of rocks in actual geological environments are complex and diverse. However, the basic principles of this model, such as the kernel function approximation and particle approximation in the SPH method, as well as the introduced concepts of the fracture marker к, stress particles, and “live and dead particles”, do not rely on the specific assumption of homogeneous materials. For rocks with different material properties in different geological environments, only by adjusting the corresponding material parameters (such as elastic modulus, tensile strength, Poisson′s ratio, cohesion, internal friction angle, etc.), can the model be applied to a certain extent for simulation analysis. For example, in the geological condition of weak surrounding rock, the rock material parameters can be set to lower strength values to reflect its poor bearing capacity, while for hard rocks, higher parameter values can be adopted. In this way, the model can adapt to the changes in the properties of different geological materials and thus simulate and predict the tunnel excavation damage in different geological environments. (2) In terms of geological structure complexity, in addition to bedding structures, there are various complex geological structures in the actual geological environment, such as faults and folds. For the geological environment with faults, the fault can be regarded as a special discontinuity surface. By reasonably setting the position, occurrence, and mechanical properties of the fault in the model (such as reducing the strength parameters at the fault and increasing its permeability, etc.), the influence of the fault on the tunnel excavation damage can be simulated. In the folded geological condition, due to the uneven stress distribution caused by the bending deformation of the rock strata, the stress boundary conditions and the parameters of the rock strata in the model can be adjusted according to the shape of the fold and the mechanical properties of the rock strata to study the influence of the fold on the stability of the tunnel. Although this requires more in-depth geological surveys and parameter determination, the framework of this model has the possibility to handle these complex geological structures and provides a basis for studying the tunnel excavation problems in different geological structural environments. (3) In terms of different tunnel types, this study takes a circular tunnel as an example for simulation analysis, but the model method can be extended to other shapes of tunnels, such as rectangular, horseshoe-shaped, etc. For differently shaped tunnels, the main influence lies in the change in the excavation boundary conditions. When applying the model, the excavation area and boundary conditions need to be redefined according to the actual shape of the tunnel. For example, for a rectangular tunnel, the excavation sequence and stress release method of the four boundaries need to be accurately set; for a horseshoe-shaped tunnel, the stress concentration characteristics of its unique curved boundary and the crown and invert need to be considered. By adjusting these boundary conditions and corresponding calculation parameters, the model can simulate the excavation process of differently shaped tunnels and analyze their damage evolution mechanisms.

## 5. Conclusions

(1) The fracture marker *к* that can reflect the failure of SPH particles is introduced, which can realize the simulation of the progressive failure process of rock.
(2) The definition of “stress particles” under the SPH framework is introduced, and at the same time, the concept of “live and dead particles” is defined, which can realize the simulation of tunnel excavation damage under confining pressure.
(3) The simulation of the tunnel excavation damage process without joints is carried out. Tensile–shear composite failure occurs in the 2 o′clock and 8 o′clock directions around a tunnel with a small range, while shear failure occurs in the 4 o′clock and 10 o′clock directions around a tunnel with a large range. The simulation results are similar to the actual engineering failure surface, which verifies the rationality of the algorithm.
(4) The tunnel excavation damage process under different bedding dip angles is simulated. First, a circle of excavation damage zones is generated around the tunnel. Obvious shear cracks are generated at the place where the joint is tangent to the tunnel. The damage degree is the largest when *α* = 30° and the smallest when *α* = 45°.
(5) The tunnel excavation damage process under different stress ratios is simulated. First, damage is generated around the tunnel. Then, the damage continuously evolves. Finally, a damage zone surrounding the tunnel is formed on the inner side of the two joints tangent to the tunnel, leading to the failure of the model. With the increase in the stress ratio, the excavation damage degree of the tunnel gradually increases.
(6) However, it should be noted that in the actual rock fracture process, the occurrence and development of fractures are extremely complex physical processes, affected by a variety of microscopic and macroscopic factors. Although the fracture marker к can effectively capture the failure of particles at the macroscopic level in our current simulation, the consideration of these microscopic structural factors is relatively limited. This means that when applying the simulation results to actual engineering, caution may be required, especially when dealing with engineering problems that are sensitive to the microstructure of the rock.

## Data Availability

Date will be available upon reasonable request.

## References

[1] Duan S., Jiang X., Jiang Q. (2023). Theoretical solution and failure analysis of water pressure on lining of deep-buried non-circular hydraulic tunnel based on the equivalent hydraulic radius method. Eng. Fail. Anal..

[2] Sun B., Wang P., Deng M. (2024). Seismic performance assessment of hydraulic tunnels considering oblique incoming nonstationary stochastic SV waves based on the generalized PDEM. Tunn. Undergr. Space Technol..

[3] Ma Z., Zhang J., Ren X. (2022). Calculation of tunnel lining structure based on boundary value method and finite element method. Water Power.

[4] Xiang X., Ren X., Zhang J. (2022). Stability analysis of surrounding rock of deep-buried tunnel based on equivalent seepage force method. Water Power.

[5] Hou J., Zhang C., Shan Z. (2011). Rockburst characteristics and prevention measures of deep-buried diversion tunnel of Jinping II Hydropower Station. Chin. J. Undergr. Space Eng..

[6] Wang J., Zheng J. (2021). Research and practice on major key technologies of Jinping I Hydropower Station project construction. J. Hydraul. Eng..

[7] Wu S., Wang G. (2011). Rock mechanical problems and optimization for the long and deep diversion tunnels at Jinping II hydropower station. J. Rock Mech. Geotech. Eng..

[8] Lei M.F., Peng LM Shi C. (2015). Model test to investigate the failure mechanisms and lining stress characteristics of shallow buried tunnels under unsymmetrical loading. Tunn. Undergr. Space Technol..

[9] Wei L. (2015). Similarity simulation test of fluid-solid coupling in subsea tunnel. J. Cent. South Univ. Sci. Technol..

[10] Yang S.Q., Chen M., Fang G. (2018). Physical experiment and numerical modelling of tunnel excavation in slanted upper-soft and lower-hard strata. Tunn. Undergr. Space Technol..

[11] Zhang D.M., Huang H.W., Hu Q.F. (2015). Influence of multi-layered soil formation on shield tunnel lining behavior. Tunn. Undergr. Space Technol..

[12] Li S., Wang D., Wang Q. (2013). Development and application of large-scale geomechanical model test system for deep thick top coal roadway. J. China Coal Soc..

[13] Li W., Li S., Wang Q. (2013). Model test study on deformation and failure mechanism of surrounding rock of deep thick top coal roadway. Rock Soil Mech..

[14] Chen J., Chu K., Wang T. (2001). Design of initial support of tunnel by convergence-confinement method. J. Xi’an Highw. Univ..

[15] Carlos C.-T., Maxwell E. (2017). The support characteristic curve for blocked steel sets in the convergence-confinement method of tunnel support design. Tunn. Undergr. Space Technol..

[16] Carranza-Torres C., Fairhurst C. (2000). Application of the Convergence-Confinement method of tunnel design to rock masses that satisfy the Hoek-Brown failure criterion. Tunn. Undergr. Space Technol..

[17] Sharan S.K. (2003). Elastic-brittle-plastic analysis of circular openings in Hoek-Brown media. Int. J. Rock Mech. Min. Sci..

[18] Sharan S.K. (2005). Exact and approximate solutions for displacements around circular openings in elastic–brittle–plastic Hoek–Brown rock. Int. J. Rock Mech. Min. Sci..

[19] Liu Z., Liu J., He W. (2024). Nonlinear seismic response and damage evolution of a mountain tunnel: Multi-scale simulation by IBEM-FEM coupled method. Eng. Anal. Bound. Elem..

[20] Xie H., He C. (2006). Analysis of excavation stability in hard rock tunnel by brittle elastoplasticity damage FEM. Tunn. Undergr. Space Technol..

[21] Zhuang Y., Cui X., Hu S. (2023). Numerical simulation and simplified analytical method to evaluate the displacement of adjacent tunnels caused by excavation. Tunn. Undergr. Space Technol..

[22] Wang W., Feng X.-T., Wang Q., Kong R., Yang C. (2024). 3D DEM simulation of hard rock fracture in deep tunnel excavation induced by changes in principal stress magnitude and orientation. J. Rock Mech. Geotech. Eng..

[23] Wang Y., Wang H., Zhao X., Tang L. (2023). Fracture of brittle material with two 3D parallel internal cracks under thermal stress: Experimental and numerical analysis. Eng. Fract. Mech..

[24] Zhou Z., Gao T., Sun J., Gao C., Bai S., Jin G., Liu Y. (2024). An FDM-DEM coupling method based on REV for stability analysis of tunnel surrounding rock. Tunn. Undergr. Space Technol..

[25] Liu B., Meng W., Zhao Z., Lin T., Zhang J. (2023). Coupled thermal-hydro-mechanical modeling on characteristics of excavation damage zone around deep tunnels crossing a major fault. Tunn. Undergr. Space Technol..

[26] Lei B., Zuo J., Massimo C., Wu G., Liu H., Yu X. (2023). Experimental and numerical investigation on meso-fracture behavior of Beishan granite subjected to grain size heterogeneity. Eng. Fract. Mech..

[27] Zhou X., Du E., Wang Y. (2022). Thermo-hydro-chemo-mechanical coupling peridynamic model of fractured rock mass and its application in geothermal extraction. Comput. Geotech..

[28] Zhou X., Zhang T. (2022). Generalized plastic ordinary state-based peridynamic model with shear deformation of geomaterials. Int. J. Rock Mech. Min. Sci..

[29] Yang Y., Xia Y., Zheng H. (2021). Investigation of rock slope stability using a 3D nonlinear strength-reduction numerical manifold method. Eng. Geol..

[30] Liu X., Hu C., Liu Q., He J. (2021). Grout penetration process simulation and grouting parameters analysis in fractured rock mass using numerical manifold method—ScienceDirect. Eng. Anal. Bound. Elem..

[31] Spetz A., Denzer R., Tudisco E., Dahlblom O. (2020). Phase-field fracture modelling of crack nucleation and propagation in porous rock. Int. J. Fract..

[32] Zhou S., Zhuang X., Zhou J., Liu F. (2021). Phase Field Characterization of Rock Fractures in Brazilian Splitting Test Specimens Containing Voids and Inclusions. Int. J. Geomech..

[33] Zhou X., Zhao Y., Qian Q. (2015). Smooth particle hydrodynamic numerical simulation of rock failure under uniaxial compression. Chin. J. Rock Mech. Eng..

[34] Zhao Y., Zhou X., Qian Q. (2015). Progressive failure processes of reinforced slopes based on general particle dynamic method. J. Cent. South Univ..

[35] Zhou X., Zhao Y., Qian Q. (2015). A novel meshless numerical method for modeling progressive failure processes of slopes. Eng. Geol..

[36] Bi J. (2016). The Fracture Mechanisms of Rock Mass Under Stress, Seepage, Temperature and Damage Coupling Condition and Numerical Simulations by Using the General Particle Dynamics (GPD). Ph.D. Thesis.

[37] Bi J., Zhou X. (2017). A Novel Numerical Algorithm for Simulation of Initiation, Propagation and Coalescence of Flaws Subject to Internal Fluid Pressure and Vertical Stress in the Framework of General Particle Dynamics. Rock Mech. Rock Eng..

[38] Bi J., Zhou X., Qian Q. (2016). The 3D Numerical Simulation for the Propagation Process of Multiple Pre-existing Flaws in Rock-Like Materials Subjected to Biaxial Compressive Loads. Rock Mech. Rock Eng..

[39] Bi J., Zhou X. (2015). Numerical Simulation of Zonal Disintegration of the Surrounding Rock Masses Around a Deep Circular Tunnel Under Dynamic Unloading. Int. J. Comput. Methods.

[40] Zhou X., Bi J., Qian Q. (2015). Numerical Simulation of Crack Growth and Coalescence in Rock-Like Materials Containing Multiple Pre-existing Flaws. Rock Mech. Rock Eng..

[41] Zhou X., Bi J. (2016). 3D Numerical Study on the Growth and Coalescence of Pre-existing Flaws in Rocklike Materials Subjected to Uniaxial Compression. Int. J. Geomech..

[42] Zhou X., Fan X., Du E. (2022). Hydromechanical coupling model for mechanical and hydraulic behavior of fractured rock mass in the framework of advanced general particle dynamics. Fatigue Fract. Eng. Mater. Struct..

[43] Zhou X., Yao W., Berto F. (2021). Smoothed peridynamics for the extremely large deformation and cracking problems: Unification of peridynamics and smoothed particle hydrodynamics. Fatigue Fract. Eng. Mater. Struct..

[44] Zhou X., Xie Y., Bi J. (2019). Numerical simulation of supershear ruptures in rock mass based on general particle dynamics. Fatigue Fract. Eng. Mater. Struct..

[45] Libersky L., Peetschek A. (1996). High strain Lagrangian hydrodynamics: A three-dimensional SPH code for dynamic material response. J. Comput. Appl. Mech. Eng..

[46] Huadong Engineering Corporation Limited, PowerChina, Institute of Rock and Soil Mechanics, Chinese Academy of Sciences (2013). General Research Report on the Generation Mechanism, Laws and Prevention and Control Measures of Rockburst in the Diversion Tunnel of Jinping II Hydropower Station.

[47] Yu S., Ren X., Zhang J., Sun Z. (2023). Numerical simulation on the excavation damage of Jinping deep tunnels based on the SPH method. Geomech. Geophys. Geo-Energy Geo-Resour..

[48] Civera M., Dalmasso M., Chiaia B. (2024). Assessing the Seismic Performance of Underground Infrastructures to Near-Field Earthquakes. Int. J. Civ. Infrastruct..

